# Measuring Pro-Environmental Behavior Triggered by Environmental Values

**DOI:** 10.3390/ijerph192316013

**Published:** 2022-11-30

**Authors:** Nuryazmin Ahmat Zainuri, Norshariani Abd-Rahman, Lilia Halim, Mee Yeang Chan, Nisa Nadirah Mohd Bazari

**Affiliations:** 1 Department of Engineering Education, Faculty of Engineering and Built Environment, Universiti Kebangsaan Malaysia, Bangi 43600, Malaysia; 2 Institute of Islam Hadhari, Universiti Kebangsaan Malaysia, Bangi 43600, Malaysia; 3 Faculty of Education, Universiti Kebangsaan Malaysia, Bangi 43600, Malaysia

**Keywords:** socioeconomic status, intergenerational, pro-environmental behavior, students, climate change education

## Abstract

Pro-environmental behavior in addressing climate change is influenced by multi-dimensional factors—knowledge, values, intention and sociodemographic background. Correlational studies between environmental values and environmental behaviors have not been able to determine values or behaviors that need to be given priority in future interventions. Therefore, this study firstly determined the environmental values and pro-environmental behavior that are easy or difficult to embrace by 152 respondents with low socioeconomic background. Secondly, we identified the extent pro-environmental behavior is triggered by environmental values. This survey study employs the Rasch analysis model. The respondents had difficulty in associating themselves with biospheric values however readily demonstrated consideration toward altruistic values, especially related to concerns for future generations. In terms of environmental conservation behavior, the respondents were not willing to relinquish comfort easily, such as giving up self-driving and taking public transportation or reducing usage of electricity. In addition, adults of low socioeconomic background find it difficult to endorse statements such as getting involved in campaigns related to environmental conservation. Thus, younger family members must be educated about conservation behaviors such as environmental campaigns commonly offered at schools, and these youngsters can be encouraged to extend their role by educating their parents.

## 1. Introduction

Climate change that has led to global warming, pollution and threats to biodiversity worldwide is primarily brought about by human activities which have accelerated because of rapid industrialization and urbanization [1]. This state of affairs is causing a pitfall to ecosystems, community health and society’s quality of life [1,2,3]. Since the root of the problem is human behavior, a major change in behavior toward the environment is therefore essential [4]. Although the percentage of people who are concerned about environmental issues is high, there is a gap in behavior as many still fail to take appropriate actions to mitigate climate change [5,6].

Studies that associate pro-environmental behavior with sociodemographic factors show inconsistent and conflicting findings [7], especially in terms of socioeconomic status (SES). Valdez et al. [8] argued that students from low SES demonstrated low pro-environmental behavior. However, Valdez’s finding is contradictory to the finding of Wan Hussain et al. [7], where the latter found that individuals with low socioeconomic status practiced good electricity saving behavior. This shows that environmental and financial factors contribute to the practice of sustainable consumption. Even though these two studies focus on children or adolescents as a targeted group, in terms of intergenerational learning, parents can influence their children’s environmental behavior and vice versa [9].

Each person puts in different effort in protecting the environment. Previous researchers have stated that personal values are able to explain or identify the lack of pro-environmental actions taken by individuals [10,11]. Previous studies [12,13] examined environmental values as an aggregate concept and not based on individuals. However, Ajzen [14,15] through the theory of planned behavior states that behavioral values are a better predictor compared to environmental values in general.

There are generally three environmental values to be considered in understanding the value of environmental care behavior, namely egoistic, biospheric and altruistic values [16,17,18]. Egoistic values emphasize one’s own well-being [19], while biospheric values emphasize the environment [10], and altruistic values emphasize the interests of others or future generations [16]. Lönnqvist et al. [20] in their study showed that an individual does not necessarily act based on their personal values but may also be influenced by the values of the society and the environment. Therefore, it is crucial to study the values that drive a nation’s environmental awareness before implementing environmental policies [21]. 

The "value–action gap" view is well-documented because there are numerous other elements that can either encourage or prevent pro-environmental behavior [22]. According to the planned behavior theory, intention is the strongest predictor of pro-environmental behavior. In this regard, the relationship between socioeconomic factors, environmental values orientation and pro-environmental behavior needs to be examined using methodological and analytical methods that are able to illuminate the association of the three components (sociodemography, environmental values and pro-environmental behavior) towards proposing intervention strategies according to priorities.

## 2. Literature Review

Behavior toward the environment is complex and influenced by various factors. The planned behavior theory [14] and the theory of value-belief-norm [16] are commonly used in predicting behaviors. According to the theory of planned behavior, intention is a prominent factor in predicting pro-environmental behavior [14]. Intention can be predicted from attitudes toward the behavior, subjective norms and perceived behavioral control. Intentions and perceptions of behavioral control can influence actual behavior directly [14]. On the other hand, the theory of value-belief-norm postulates that values influence pro-environmental behavior via pro-environmental beliefs and personal norms. In other words, individuals engage in pro- environmental behaviors because they believe in and are aware of adverse consequences of environmental problems for themselves, other people and the biosphere [16].

The two existing environmental theories proposed three main factors that encourage a person to show behavior towards the environment, namely internal, environmental, and sociodemographic factors [6]. Internal factors are associated with held values, attitudes, and self-efficacy, while environmental factors involve facilities, local culture, community, and environmental campaigns. Meanwhile, sociodemographic factors consist of age, gender, education level, social class and household income [23]. Values are guiding principles in life that serve as a person’s inspiration for taking action to protect the environment [22]. The three common and fundamental environmental values are altruistic, biospheric and egoistic [16,17,18].

Based on a model of values by Schwartz [18], biospheric focus on nature consists of values of protecting the environment. Previous research found that biospheric values influence environmental behavior [24,25]. Altruistic values, on the other hand, are related to the welfare of those in the larger society, consist of the values of equality, social justice, peace on earth and being helpful. Meanwhile, egoistic values focus on self-enhancement and consist of being ambitious, influential, wealthy, having authority, social power and achievement. The egoistic and altruistic values are opposite to each other, in which altruistic values emphasize concern for the welfare and interests of others (universalism, benevolence), while egoistic values emphasize the pursuit of one’s own interests [18,22,26].

Values are influenced by the relationship between sociocultural, education and environmental contexts [27,28]. As suggested by Moser and Kleinhückelkotten [23], sociodemographic factors (income, household size or geographic location) strongly determine people’s lifestyles that lead to environmental values and behavior that have an impact on the environment. Rachmatullah et al. [27] found in their study that residents in developing countries such as Indonesia have higher egoistic values compared to those in developed countries such as Korea.

Individuals adopt the environmental behavior that is convenient for them, such as self-driving rather than taking public transport [29,30]. Similarly, individuals with low socioeconomic status tend to act in relation to the environment with financial considerations in mind [31,32]; an example of this is in terms of energy use [33,34]. However, Abrahamse and Steg [35] in their study found that income has a great influence on the reduction of energy consumption. This suggests that low socioeconomic status linked to low education has an impact on environmental awareness, where those within this group tend to display lower awareness of the environment [35].

Research evidence suggests that younger generations engage in more positive attitudes toward environmental issues compared to the older generations; however, they tend to be hesitant in following through with the behavior [36]. It is further suggested that the youngsters are implausible instigators of pro-environmental behavior changes, but their parents seem more geared up for this pursuit. Hence, the parents’ action towards pro-environmental behavior directly and contingently influences their children to act the same. Furthermore, parents with higher socioeconomic status appear to be the most likely to perform pro-environmental behavior as they have access to educational opportunities and understand the values better and thus, have more potential to inspire their children [9,36]. In this regard, intergenerational influence of attitudes, values and behavior towards the environment would proliferate from the parents to the children.

Evidence shows that environmental values are linked to environmental behavior. Research findings by Torkar and Bogner [4] on primary and secondary school students indicate that altruistic, egoistic and biospheric values have a positive and strong correlation with environmental preservation. Adolescent individuals have been shown to report that egoistic values such as personal well-being, health, and personal development highly influence their pro-environmental behavior [37]. However, Howell and Allen [22] in their study demonstrated that altruistic and biospheric values equally contribute to concern about the environment despite egoistic values showing a negative relationship with pro-environmental behavior. Biospheric values are related to a comprehensive set of pro-environmental behaviors such as sustainable consumption, environmental activism, preference for green restaurants and donating money to environmental organizations [25].

A study in China found that altruistic values influenced pro-environmental behavior among the study’s participants, but their socioeconomic status did not influence pro-environmental behavior, especially in donating for environmental preservation [38]. Similarly, Waris et al. [39] conducted a study in Turkey that found that altruistic values strongly influenced the consumption of environmentally friendly appliances. Similarly, Ivanova et al. [40] in their study found that the purchase of energy-efficient goods was higher among residents with high socioeconomic status. However, the increase in income also increased the intake of meat and dairy products that have a greater impact on the environment, especially climate change [40].

Culture or traditional values also influence values toward pro-environmental behavior [41,42]. Thus, previous studies have found that there is a difference between people from the East and those from the West in terms of their values toward the environment [43]. Punzo et al. [44] found that in Italy and the United Kingdom, the perceived values of the people displayed a direct relationship with pro-environmental behavior. Conversely, for the people in Germany and France, values were found to be less indicative of behavior as their European cultural identity is more prominent. Additionally, Milfont et al. [45] in their study found that biospheric values positively predicted pro-environmental behavior for ethnic Europeans in New Zealand, while egoistic values predicted pro-environmental behavior negatively. In contrast, for people of Asian ethnicity in New Zealand, both biospheric and altruistic values positively predicted pro-environmental behavior. A comparative study involving Japan, Thailand and the Philippines discovered that a country’s environmental values are in line with its traditional values [46]. Despite the considerable number of studies available, studies on the values of the environment related to socioeconomic factors in the context of Asian countries are still limited [27].

Pro-environmental behavior is seen to vary with the environmental values adopted. Behavior toward the environment through consumerism is related to egoistic values [27]. Chua et al. [47] in their study found that biospheric and egoistic value orientations influenced customers’ readiness to pay expensive prices in green restaurants; however, this was not the case with altruistic values. In contrast, past studies have shown behavior related to climate change mitigation to be influenced by altruistic values such as thinking about the impact on the poor and sustainability for future generations [22,37]. Thus, there appears to be a complex link between environmental values and pro-environmental behavior.

Hence, there is a need to explore further the link between the values and intentions of pro-environmental behavior by considering the respondents’ demographic background. In the context of this study, the focus was on people who are categorized in the low socioeconomic status group. This group was chosen as previous studies have generally found that individuals with low socioeconomic status have low pro-environmental behavior [48]. Weckroth and Ala-Mantila [33] who examined value orientation in the context of European countries found those in the lower income deciles reporting greater engagement with energy saving behavior despite reporting weaker pro-environmental norms. Having better understanding of the environmental values held by this target group would therefore allow interventions to be carried out more effectively through increasing pro-environmental behavior among people with low socioeconomic status, especially in and outside the school education context.

In addition, to identify links between environmental values and pro environmental behavior, it is also imperative to identify environmental values that are easily adopted by a person. An individual adopts environmental values depending on the situation they are facing [49]. Thus, calibrating one’s values on a single continuum indicates what environmental values need to be addressed and given priority. A similar argument is put forth for pro-environmental behavior [50], where adoption of a particular behavior is associated with temporarily benefitting the individual; thus by calibrating one’s pro-environmental behavior on a single continuum, again, one is able to give priority to the behavior that needs to be addressed. 

In other words, through the calibration process, one can identify the critical environmental values and pro-environmental behavior that require attention to be environmentally responsible individuals. Such calibration can be accomplished by adopting the Rasch analysis approach. Similarly, the Rasch analysis approach provides an alternative perspective in exploring the link between environmental values and pro-environmental behavior. Thus, the main contribution of this study is to provide an alternative approach to methodology that enhances the understanding of environmental values and pro-environmental behavior that contribute towards individuals of those from the lower socioeconomic background. 

Therefore, this study addressed the two complementary research gaps. Firstly, it sought to investigate the environmental values and intentions of easily adopted pro-environmental behaviors that require critical attention. Secondly, the study aimed to explore the association between environmental values and the intention for pro-environmental behavior through the Rasch measurement model. 

The research questions are:

1. What are the environmental values deemed easy and difficult to adopt by the respondents from low socioeconomic background?
2. What are the pro-environmental behaviors deemed easy and difficult to rate by the respondents from low socioeconomic background?
3. How are the environmental values linked to the pro-environmental behaviors as mapped out by the Rasch analysis?

Based on research questions, the hypotheses are:

1. Environmentally responsible individuals are highly related to the environmental values orientation.
2. Environmentally responsible individuals are highly related to the intention for pro- environmental behaviors.
3. Pro-environmental behaviors are affected by values orientation.

## 3. Methodology

### 3.1. Research Design

This study employed the survey method. Survey method is relevant for this study as it provides a baseline data to a study that is beginning to embark investigating the environmental values and behavior towards environment conversation among those from the low socioeconomic group. 

### 3.2. Sample

The convenience sampling technique is used in this study as a baseline study. More specifically, the study involved a total of 152 respondents from Kuantan district in Pahang, Malaysia. The respondents and their demographic profile (see Table 1) consisted of 91 women and 61 men whose age ranged between 21 and 51 years old and above. All the respondents had a household income of less than MYR 3899, where they belonged to the category of households of lower socioeconomic status or known as the B40 (the lowest 40% of income group in Malaysia). Based on the rate released in 2018 during the Budget 2019 report, the B40s are households earning an average monthly income below MYR 3860, which also includes those with average incomes of less than the Poverty Income Line of MYR 950 per month [51]. 

### 3.3. Instrument

The study instrument was a survey consisting of three parts. Part A contained demographic-related questions (3 items), while Part B was on environmental values (12 items), and Part C was on pro-environmental behavior (22 items). The instrument for environmental value variables was adapted from [52]. Items were measured using a five-point Likert scale (1: Not Important; 2: Slightly Important; 3: Moderately Important; 4: Important; 5: Very Important). Meanwhile, the instrument for behavioral intention toward environmental conservation was adapted and modified from various studies [53,54,55,56]. A five-point Likert scale (1: Strongly Disagree; 2: Disagree; 3: Slightly Agree; 4: Agree; 5: Strongly Agree) was used for measuring the items. The research instrument underwent the process of checking for content validity and was evaluated by experts in environmental education and climate change. Additionally, the instrument was also tested in a pilot study which involved 35 respondents. Appendix A presents the complete questionnaire.

### 3.4. Data Collection Procedure

This study used a Google Form survey to obtain primary data. The Google Form link was shared with the respondents through WhatsApp, Telegram and Facebook. The respondents were informed of the purpose of the study and data gathered was mainly for research purposes and reported as aggregate data and is anonymous. 

### 3.5. Data Analysis

#### 3.5.1. Rasch Model 

The Rasch technique has attracted researchers to validate and evaluate constructed items in creating a good survey [57,58]. In this study, the Rasch measurement model was used to identify the relationship between environmental values and the intention for behavioral environmental conservation (among people in the low socioeconomic group). In the model of this study, persons represent the respondents with low socioeconomic status in the district of a state in Malaysia and the items represent the environmental value orientation and intention for environmental conservation behavior. The distribution of persons and items was measured using a measuring standard known as “logits”. Rasch models transform the raw data and allow conjoint measurement of people and items in the same scale. This is accomplished using the item map, also known as the Wright map, where the person and items are plotted on the same logit scale or ruler. Bond and Fox [59] explained that the Rasch model allows the researcher to identify two aspects: (1) the items that are easily agreed or disagreed upon by the person (respondents), and (2) a person with high ability may be more agreeable with all the items, which means a high-ability person finds it easy to endorse the survey. In this study, it was assumed that the respondent’s espoused environmental values should relate to the behavior that contributes to environmental conservation. 

#### 3.5.2. Reliability and Separation Index

Reliability and separation index are used to demonstrate the accuracy of the instrument used in a study [60] so that the instrument can consistently and accurately measure what it is meant to measure. Based on Bond and Fox [59], reliability values between 0.81 and 0.90 are considered good, while values between 0.91 and 0.94 are considered very good, and those greater than 0.95 are regarded as excellent. Meanwhile, for the separation value, a good value of separation is between 3 and 4, while a very good value is between 4 and 5. Additionally, based on Linacre [61], a separation value that is greater than 2 is considered good.

Table 2 shows the summary statistics for the respondents (persons) and the items. The Cronbach alpha value was 0.92, which means the interaction between the respondent and items as a whole is in a good category. The mean square (MNSQ) is a chi-square statistic which is used to compute the item fit. From Table 2, the statistics show the mean for both infit MNSQ and outfit MSSQ for respondents and items are close to 1.0, which indicate the data had shown acceptable fit to the model. The reliability value of the respondent was 0.81, with separation value of 2.08, indicating that the respondents had a small range of ability in that the items could be categorized into 2-level spread. Meanwhile, the item reliability value was 0.94, which showed that the items on factors influencing environmental behavior are good and acceptable for use in this research. The item separation value was 4.12, which indicated that the items could be categorized into four difficulty levels.

#### 3.5.3. Item Measure and Unidimensionality

The raw, unexplained variance (total) of the standardized residual variance in eigenvalue units measures item dimensionality. In this study, the eigen value obtained was 34.0 with an observed percentage of 63.0%, and according to Linacre [61], a variance of more than 40% indicates items to be unidimensional. This shows that the items used in this survey where the items represent the environmental value orientation and intention for environmental conservation behavior are relevant to the aim of this research. Thus, the next step was to determine the item validity of the developed survey items.

In the Rasch analysis model, the criteria used for determining item validity are point measure correlation (PTMEA), outfit mean square (MNSQ) and outfit Z-standard (ZSTD). Table 3 shows the item polarity based on PTMEA where this value is used to ensure the items are parallel in measuring the intended construct [61,62]. Based on Table 3, the PTMEA values are noticeably positive (0.2 and above), indicating that the items can measure what they are supposed to measure [59]. 

To validate the items, the acceptable regions are as follows: 0.4 < PTMEA < 0.8, 0.5 < MNSQ < 1.5 and −2 < ZSTD < 2. Analysis obtained from Table 3 shows that all the items are valid except for item numbers B2, B10 and C1. However, these three items were not deleted since the infit MNSQ was in the acceptable range [61]. Overall, the analysis showed that the items are suitable for the aim of this study which sought to examine intention toward environmental conservation behavior.

## 4. Results

**Figure 1:**
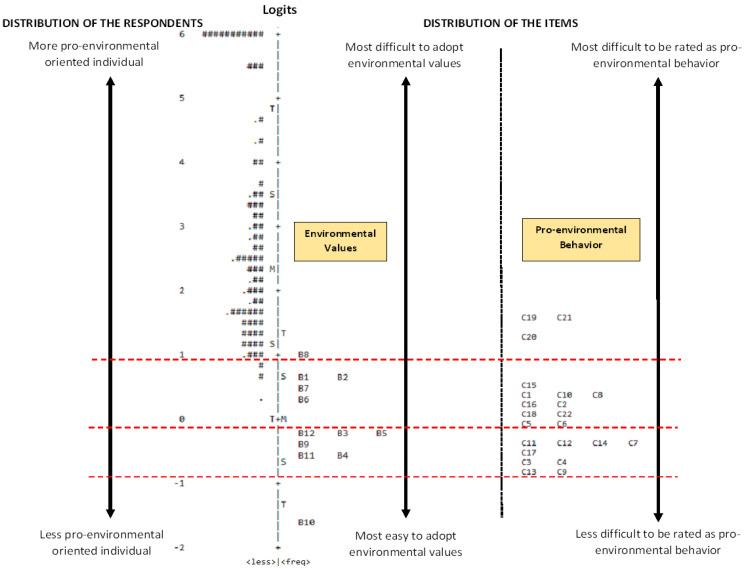
Item map for the constructs of environmental values and pro-environmental behavior for environmental conservation. Each ‘#’ represents two respondents and ‘.’ represents one respondent.

Figure 1 shows the map of 12 items (environmental values) and 22 items (pro- environmental behavior) obtained from the Rasch analysis. All items are on a single continuum (ruler) and all items have been calibrated on that single ruler. The item that respondents have the most difficulty agreeing to has the highest logit measure. In contrast, the item that respondents can most easily agree with has the lowest logit measure and is located at the bottom of the map. In addition, the person’s mapped item may provide a clearer picture of the respondent’s ability and the item’s difficulty. Overall, based on Figure 1, the ability of the respondents was in between logit −0.69 and 6.35. This map describes that most of the individuals had high ability in answering the questionnaire items. In other words, most of the respondents found it easy to answer all the questions

As shown on the right-hand side of Figure 1, the items related to environmental values are ordered from the “Most easy to adopt environmental values” to the “Most difficult to adopt environmental values”. The right hand side of Figure 1 also displays the items related to pro-environmental behavior, which are ordered from “Less difficult to be rated as pro-environmental behavior” to “Most difficult to be rated as pro-environmental behavior”. 

The left side of Figure 1, matches the level of pro-environmental-oriented individuals, whereby the respondents are ordered from the “Less pro-environmental-oriented individual” to “More pro-environmental-oriented individual”. The more pro-environmental-oriented individual is an individual who adopts easily to the environmental values that are of biospheric nature and demonstrates pro-environmental behavior commensurate with that associated value. In contrast, the less pro-environmental-oriented individual is an individual who easily adopts the egoistic environmental value and to some extent the altruistic environmental values. Consequently, the individual demonstrates the pro-environmental behavior that aligns with those values. 

The distribution of items as a result of the Rasch analysis is shown in Figure 1 for environmental values: the items can be visualized to follow four levels of distribution and each distribution is bound by the horizontal dotted line. Similarly, the distribution of items for pro-environmental behavior is visualized to have three levels of distribution, also bound by the horizontal dotted line. Based on Figure 1, the distribution of items is rated as follows: (1) above the logit value of 1.0 is seen as most difficult, (2) between logit values of 1.0 and 0.0 is seen as difficult, (3) between logit values 0.0 and (−1.0) is seen as easy and (4) below the logit value (−1.0) is seen as the easiest to adopt and rate the environmental values and pro-environment behavior by the respondents.

Q1. What are the environmental values deemed easy and difficult to adopt by the respondents from low socioeconomic background? 

The map (Figure 1) showed that item B8 for environmental values, which was located at the top of the scale, was placed in the top most of the single continuum with logit value above 1.0, where the item corresponded with the values of consequences of environmental issues for birds (biospheric values). The respondents found it most difficult to associate themselves with this statement, that is, to adopt these environmental values.

For environmental values, items B1, B2, B7 and B6 were distributed and fall at the same level and with logit values between 1.0 and 0.0 (see Figure 1), showing that values concerning own self (B1), everyone (B2), people in my community (B7), and living organism (B6) were difficult to adopt. In other words, those environmental values were lacking among the respondents. It suggests that the environmental values are weak in oneself, members outside the circle of the family, and towards nature itself among those of lower socioeconomic background.

Items B12, B3, B5, B9, B11 and B4 were distributed in the same continuum of the single ruler, with logit values below 0.0. All the environmental values in this category focused on a person’s own health, future, children and the environment (plants and animals) and were easy to adopt by the respondents. This means that the respondents were more focused on their own selves and their close family members. In particular, for egoistic values, respondents felt that environmental problems may affect their future (B9), life (B5) and health (B3). 

Based on Figure 1, the respondents found easiest to adopt the environmental values, which is an altruistic value (B10), which is the only item at the most bottom of the single continuum. 

Q2. What are the pro-environmental behaviors deemed easy and difficult to rate by the respondents from low socioeconomic background?

For the pro-behavioral behaviors, the distribution of items C19, C21 and C20 was visualized (see Figure 1) as a set of behaviors that the respondents found most difficult to rate as pro-environmental behavior in themselves. To conserve the environment, the respondents’ intention to behave in relation to the three items was most difficult. The respondents found it difficult to consider reducing self-driving and using other methods of transportation (C19), reducing phone charging frequency to reduce electric consumption (C20) and considering cycling or walking if the travel distance is less than 5 km (C21). Items C15, C1, C10, C8, C16, C2, C18, C22, C5 and C6 were distributed in the same range of the calibration and thus situated at the same level, are difficult to rate by the respondents. The respondents showed low intention in considering having a shorter bath time to reduce water consumption (C15), buying packed products (C1), participating in awareness on energy usage campaigns (C10) and reducing the usage of air conditioning to save energy (C8). 

The respondents also showed low intention in reducing consumption of disposable products (C16), buying products that are packed or made from recycled materials (C2), considering buying fuel-efficient vehicles (C18), supporting or getting involved in any related campaigns regarding environmental conservation (C22), considering buying organically grown products (C5) and buying products in packaging that is biodegradable (C6).

The next distribution of items (see Figure 1) visually showed the respondents intention with regard to pro environmental behavior: items C11, C12, C14, C7, C17, C3, C4, C13 and C9, and these pro-environmental behaviors were rated easy by the respondents. Specifically, the respondents would consider using recycled paper (C11), biodegradable garbage plastic (C12), refillable water bottles (C7) and reusable bags for shopping (C17). The respondents would also consider buying products that do not endanger the environment (C3), using energy saving light bulbs (C4), buying energy efficient home appliances (C9) and using food packaging that is eco-friendly (C13) for environmental conservation. 

Q3. How are the environmental values linked to the pro-environmental behaviors as mapped out by the Rasch analysis?

An important result to note in Figure 1 is that the distribution of a set of items related to environmental values are aligned to the distribution of a set of items of pro-environmental behavior. The first level of distribution showed that the biospheric values (B8)—concern for birds—was most difficult to adopt by respondents, and these values corresponded to the pro-environmental behavior that respondents also found most difficult to rate—reducing self-driving and using other methods of transportation (C19), reducing phone charging frequency to reduce electric consumption (C20) and considering cycling or walking if the travel distance is less than 5 km (C21).

The second level of distribution that is the environmental values (concern for own self to concern for living organism) that the respondents found difficult to adopt is mapped to pro-environmental behaviors that lead to sustainable consumption (e.g., consumption of disposable products (C16) and buying products that are packed or made from recycled materials (C2)). 

The third level of distribution reflects the environmental values easily adopted among the respondents which revolve around oneself, family and future generations, whose values are laden towards egoistic and altruistic values. The pro environmental behaviors that corresponded to the values revolve also around sustainable consumption (using recycled paper (C11), buying energy efficient home appliances (C9) and using food packaging that is eco-friendly (C13)). 

In the level of item distribution, the easiest environmental value adopted by the respondents was item B10, which is an altruistic value. Most of the respondents were concerned about the consequences of environmental problems for future generations. However, this particular value has no direct link to any of the behavioral intentions towards environmental conservation.

## 5. Discussion

The distribution of environmental values and pro-environmental behaviors above the logit values above 0.0 is seen to be the most difficult and difficult values and behaviors to be adopted and rated by the respondents. Thus, these values and behaviors need to be given priority in intervention programs so that those values and behaviors are inculcated.

In relation to environmental values, the biospheric value focusing on the consequences of environmental issues for birds was most difficult to endorse. Environmental values toward members outside the circle of the family and toward nature itself were difficult to endorse. Adopting environmental values that reflect concern for oneself and immediate family members and their futures was easy to adopt among the respondents. In other words, egoistic values are easily adopted, as argued by Rachmatullah et al. [27], and these values are highest among developing countries. 

In relation to pro-environmental behaviors, the respondent found to be most difficult when it requires them to opt for self-driving and using other methods of transportation, the respondents still find it difficult despite of the improvement in the sector of public transport over the years, and it is still inefficient to discourage the public from self-driven [29]. In addition, the study of Steg et al. [30] found that respondents preferred to drive private vehicles compared to taking public transport because it is more independent, convenient, comfortable, and flexible; additionally, self-driving was also considered more enjoyable and able to meet the respondents’ self-satisfaction. In the context of Malaysia, the reluctance of opting to walk or cycle could be attributed to the hot weather in Malaysia and perhaps regardless of socioeconomic status as well.

At the same time, the respondent found that the items that require them to reduce phone charging frequency and electrical consumption is difficult too. This could be rationalized by the importance of the mobile phone, which has become an indispensable device in people’s daily life nowadays. The importance of smartphone mobile devices is not only limited to simplifying the communication process; it has also simplified the practice of information retrieval, especially for problem solving in humans’ day to day activities in life. Such a scenario has made this technology an indispensable item for its users, who depend on and use this technology frequently. The reliance on this technology is one of the main barriers for the respondents in this present study to consider reducing phone charging frequency. Such conservation behavior relates directly and indirectly to consumption patterns of energy usage among the respondents. However, due to its implication to the environment, high energy consumption in many countries globally, including in Malaysia, has become a major concern [63,64].

The result of this study also found that the level of desire for environmental conservation behavior and the environmental values that support pro-environmental behavior among the respondents is low. Van der Werff et al. [30] argued that biospheric values are closely related to the individual’s environmental identity and subsequently influence the preferences, wishes and behavior of the individuals toward the environment. According to Martin and Czellar [24], the stronger the individual’s relationship with the environment, the stronger the orientation of biospheric values, which in turn influences pro-environmental behaviors. This indicates that an individual’s environmental identity plays a role in the formation of biospheric value orientation. In other words, these values and activities are difficult for those of low socioeconomic background to endorse or comply with.

Thus, the finding suggests that families with low socioeconomic status in the study have the awareness and desire to practice environmental conservation by saving energy consumption at home. In a study conducted by Yohanis [34], it was found that 70–80% of the household participants took energy efficiency measures daily while 20–35% of the households wanted to engage in energy saving practices; however, they reported that cost was a major barrier for them. At the same time, the respondents who were concerned for themselves and their immediate family members and also for their future (egoistic and altruistic values) demonstrated sustainable consumption behavior despite their financial capacity. Thus, the egoistic and altruistic values and not only the biospheric values as argued by van der Werff, Steg and Keizer [25] can also drive pro-environmental behaviors leading to sustainable consumption, as shown in this study.

As shown in the study [31], respondents of higher socioeconomic status tend to show more willingness to conduct household pro-environmental behavior through their financial capacity to manage the cost incurred for such practices. Hence, it has been argued in past studies that people of low socioeconomic status are more likely to be concerned with meeting basic needs and obtaining pleasures in their comfort zone and, as such, these often prevent them from paying attention to environmental issues [32]. On the other hand, Grønhøj and Thøgersen [36] found that parents with higher socioeconomic status were most likely to perform pro-environmental behavior as they have access to educational opportunities and understand the values better and thus have more potential to inspire their children. In addition, Jia et al. [9] argue that parents have a crucial role to participate with their children in environmental issues to ensure intergenerational transmission of pro-environmental behaviors. Based on the current study’s findings, the intergenerational transmission of pro-environmental behaviors needs to be from the students to the parents who are of low socioeconomic background. Students in schools are often exposed to environmental awareness and experiences that could be shared with their parents [28].

## 6. Conclusions

This study sought to investigate the environmental values and environmental behaviors of those from a low socioeconomic background. The findings revealed that in terms of environmental values, the respondents had difficulty in associating themselves with biospheric values that do not have a direct impact on their family or everyday situation, such as caring for birds. The respondents, however, readily demonstrated consideration toward altruistic values such as concern for future generations. In terms of environmental conservation behavior, the respondents were not willing to relinquish comfort easily, such as giving up self-driving and taking public transportation or reducing usage of electricity. It was also found that the environmental values exhibited were commensurate with the environmental conservation behavior.

This study has highlighted the environmental values and conservation behaviors that would be easily adopted by the respondents who were of low socioeconomic background. It was found that adults of low socioeconomic background find it difficult to endorse statements such as getting involved in campaigns related to environmental conservation. An implication of such finding is for the younger family members to be educated on conservation behavior through such campaigns, which are commonly offered at schools, and then getting these youngsters to extend their role by educating their parents. Thus, future studies may want to look at the effect of intergenerational activities on environmental conservation behavior and its impact on environmental values. 

This study also found that the behavior of reducing energy consumption among those from the low socioeconomic background is difficult to endorse. The difficulty to adopt sustainable energy consumption is also related to the egoistic values espoused by the people belonging to this group, where their values center on their own comfort regardless of the betterment that can be gained by the members outside of their communities and nature itself. One implication is for policy makers from other sectors such as the housing development sector to consider green housing for those of low socioeconomic background so that energy consumption is lessened. Additionally, programs such as education through hands-on activities instead of awareness campaigns and demonstrations of initiatives that can lessen energy consumption and its impact in terms of monetary savings and conservation of the surroundings, and quality of life should be enhanced. In light of the finding, future studies may want to explore the effectiveness of such formal and informal education through systematic intervention. 

Similar to other studies, this study found that altruistic values, namely concern for future generations, are translated to pro-environmental behaviors that indirectly lead to monetary savings. Such a finding may possibly be due to the economic background of the respondents. Future studies could explore the reasons for such behavior through interviews with the respondents concerned. 

## Figures and Tables

**Table 1:** Respondents demographic profile.

Profile	Demographics	n	Percentage (%)
Gender	Male	61	40.1
	Female	91	59.9
Age	21–30 years old	42	27.6
	31–40 years old	34	22.4
	41–50 years old	40	26.3
	51 years old and above	36	23.7

**Table 2:** Summary statistics for respondents (persons) and items.

	Respondent (Person)	Items
Measure		
Mean	3.03	0.00
SD	1.86	0.69
Outfit MNSQ		
Mean	1.05	1.05
SD	0.67	0.48
Infit MNSQ		
Mean	1.06	1.01
SD	0.59	0.21
Separation	2.08	4.12
Reliability	0.81	0.94
Cronbach Alpha	0.92	

Note: SD = standard deviation; Outfit MNSQ = outfit mean square; Infit MNSQ = infit mean square.

**Table 3:** Item polarity and item fit.

Item	Measure	Infit		Outfit		PTMEA
		MNSQ	ZSTD	MNSQ	ZSTD	
B10	−1.27	0.92	−0.37	0.90	−0.15	0.30
B2	−0.34	0.96	−0.19	3.42	6.48	0.32
B9	−0.74	0.99	0.02	1.00	0.10	0.35
C14	−0.1	1.39	2.23	1.53	2.18	0.37
B4	−0.81	0.84	−0.90	0.81	−0.59	0.38
B5	−0.67	0.88	−0.66	0.83	−0.56	0.39
C4	−0.28	1.41	2.30	1.47	1.86	0.39
B12	−0.7	0.89	−0.63	0.78	−0.77	0.4
B3	−0.67	0.85	−0.85	0.80	−0.71	0.4
C3	−0.31	1.57	3.05	1.12	0.59	0.41
B11	−0.84	0.77	−1.36	0.67	−1.16	0.41
B8	−0.23	0.96	−0.22	1.18	0.82	0.43
B1	−0.36	0.85	−0.94	0.85	−0.59	0.44
C11	−0.03	1.24	1.44	1.25	1.16	0.44
C1	0.58	1.41	2.42	1.55	2.78	0.44
C9	−0.45	0.95	−0.28	0.74	−1.06	0.45
B7	−0.42	0.76	−1.55	0.77	−0.93	0.45
B6	−0.51	0.76	−1.53	0.69	−1.28	0.46
C2	0.49	1.39	2.31	1.39	2.00	0.47
C6	0.17	1.09	0.59	1.18	0.95	0.49
C13	−0.42	0.77	−1.48	0.52	−2.34	0.5
C17	−0.13	0.95	−0.25	0.68	−1.56	0.5
C12	−0.1	0.94	−0.35	0.69	−1.56	0.5
C7	−0.01	1.04	0.33	0.78	−1.05	0.5
C8	0.56	1.18	1.15	1.11	0.66	0.51
C18	0.4	1.08	0.55	1.03	0.23	0.51
C5	0.25	1.00	0.07	1.80	−1.06	0.53
C22	0.29	0.88	−0.77	0.88	−0.63	0.54
C10	0.59	1.04	0.33	0.96	−0.20	0.56
C15	0.81	0.99	−0.01	1.06	0.43	0.57
C16	0.53	0.78	−1.49	0.99	0.03	0.57
C19	1.65	0.92	−0.63	1.15	1.14	0.67
C21	1.7	1.02	0.17	1.10	0.80	0.67
C20	1.37	0.78	−1.72	0.87	−0.90	0.68

Note: MNSQ = mean square; ZSTD = standard mean square; PTMEA = point measure correlation.

## Data Availability

The data that support the findings of this study are available from the corresponding author upon reasonable request.

## Appendix A

The questionnaire on environmental values and pro-environmental behavior.

### Appendix A.1. Section A: Respondent Profile

Please tick (/) the following about yourself.

**Gender**	
Male	
Female	
**Age**	
21–30 years old	
31–40 years old	
41–50 years old	
Above 52 years old	

**Monthly Household Income**	
Less than RM2560	
RM2560-RM3019	
RM3020-RM3449	
RM3050-RM3899	
RM3900 and above	

### Appendix A.2. Section B: Environmental Values

Please circle the following statements according to your level of importance.

**1**	**2**	**3**	**4**	**5**
not important	slightly important	moderately important	important	very important

People around the world are generally concerned about environmental problems because of the consequences that result from harming nature. However, people differ in the consequences that concern them the most. Please rate the following items from 1 (not important) to 5 (very important). I am concern about the environment problems because of the consequences for ________

**Item**		**Importance Level**				
1.	me	**1**	**2**	**3**	**4**	**5**
2.	all people	**1**	**2**	**3**	**4**	**5**
3.	my health	**1**	**2**	**3**	**4**	**5**
4.	children	**1**	**2**	**3**	**4**	**5**
5.	my future	**1**	**2**	**3**	**4**	**5**
6.	all living things	**1**	**2**	**3**	**4**	**5**
7.	people in my community	**1**	**2**	**3**	**4**	**5**
8.	birds	**1**	**2**	**3**	**4**	**5**
9.	my future	**1**	**2**	**3**	**4**	**5**
10.	future generations	**1**	**2**	**3**	**4**	**5**
11.	animals	**1**	**2**	**3**	**4**	**5**
12.	plants	**1**	**2**	**3**	**4**	**5**

### Appendix A.3. Section C: Intention towards Pro-Environmental Behaviour

Please circle the following statements according to your level of agreement.

**1**	**2**	**3**	**4**	**5**
Strongly disagree	Disagree	Slightly agree	Agree	Strongly agree

**No**	**Item**	**Level of Agreement**				
1	I will buy products that are packed or made from refillable container.	1	2	3	4	5
2	I will buy products that are packaged or made from recycled materials.	1	2	3	4	5
3	I will buy products that do not harm the environment.	1	2	3	4	5
4	I will use energy saving lights.	1	2	3	4	5
5	I will buy organic product.	1	2	3	4	5
6	I will purchase biodegradable packaging products.	1	2	3	4	5
7	I will buy a product from company that prioritizes the environment (with MyHIJAU logo).	1	2	3	4	5
8	I will reduce the use energy saving air condition.	1	2	3	4	5
9	I will buy energy efficient home appliances.	1	2	3	4	5
10	I will join the electrical saving awareness campaign.	1	2	3	4	5
11	I will reuse papers for making notes or printing.	1	2	3	4	5
12	I will use biodegradable trash plastic at home	1	2	3	4	5
13	I will use eco-friendly food packaging products	1	2	3	4	5
14	I will use a refillable water bottle.	1	2	3	4	5
15	I will shorten the shower time to reduce water consumption	1	2	3	4	5
16	I will reduce the use of one-time usage type container.	1	2	3	4	5
17	I will bring a reusable bag for shopping.	1	2	3	4	5
18	I will choose to buy fuel efficient vehicle.	1	2	3	4	5
19	I will drive less and use other modes of transportation	1	2	3	4	5
20	I will reduce the frequency of charging phone to reduce electricity consumption.	1	2	3	4	5
21	I will cycle or walk if the journey is less than 5 km	1	2	3	4	5
22	I will support or involve in campaigns related to environmental conservation efforts.	1	2	3	4	5
